# Integrated genomics, physiology and breeding approaches for improving drought tolerance in crops

**DOI:** 10.1007/s00122-012-1904-9

**Published:** 2012-06-14

**Authors:** Reyazul Rouf Mir, Mainassara Zaman-Allah, Nese Sreenivasulu, Richard Trethowan, Rajeev K. Varshney

**Affiliations:** 1 International Crops Research Institute for the Semi-Arid Tropics (ICRISAT), Patancheru, Hyderabad, 502 324 India; 2 Division of Plant Breeding and Genetics, Sher-e-Kashmir University of Agricultural Sciences and Technology of Jammu (SKUAST-J), Chatha, Jammu, 180 009 India; 3 Department of Biology, Faculty of Sciences, University of Maradi, BP 465, Maradi, Niger; 4 Leibniz Institute of Plant Genetics and Crop Plant Research (IPK), 06466 Gatersleben, Germany; 5 Plant Breeding Institute, University of Sydney, PMB11, Camden, NSW 2570 Australia; 6 CGIAR-Generation Challenge Programme (GCP), c/o CIMMYT, Int APDO Postal 6-641, 06600 Mexico, DF Mexico; 7 School of Plant Biology (M084), Faculty of Natural and Agricultural Sciences, The University of Western Australia, 35 Stirling Highway, Crawley, WA 6009 Australia

## Abstract

Drought is one of the most serious production constraint for world agriculture and is projected to worsen with anticipated climate change. Inter-disciplinary scientists have been trying to understand and dissect the mechanisms of plant tolerance to drought stress using a variety of approaches; however, success has been limited. Modern genomics and genetic approaches coupled with advances in precise phenotyping and breeding methodologies are expected to more effectively unravel the genes and metabolic pathways that confer drought tolerance in crops. This article discusses the most recent advances in plant physiology for precision phenotyping of drought response, a vital step before implementing the genetic and molecular-physiological strategies to unravel the complex multilayered drought tolerance mechanism and further exploration using molecular breeding approaches for crop improvement. Emphasis has been given to molecular dissection of drought tolerance by QTL or gene discovery through linkage and association mapping, QTL cloning, candidate gene identification, transcriptomics and functional genomics. Molecular breeding approaches such as marker-assisted backcrossing, marker-assisted recurrent selection and genome-wide selection have been suggested to be integrated in crop improvement strategies to develop drought-tolerant cultivars that will enhance food security in the context of a changing and more variable climate.

## Introduction

Drought is the most devastating abiotic stress affecting crop productivity, which is caused by insufficient rainfall and/or altered precipitation patterns (Toker et al. 2007). The seriousness of drought stress depends on its timing, duration and intensity (Serraj et al. 2005). The impact of drought on crop production has been evidenced as early as the beginning of the seventeenth century, known as “Sahel drought”, caused due to human intervention effects of deforestation, overgrazing and industrialization (Held et al. 2005). Increase in greenhouse emissions has resulted in altered precipitation, increase in arid land, desertification and finally reduction in crop productivity. Moreover, it has been causing global warming, which in turn is responsible for raising the earth’s surface temperature and sea water level. As of today, climate–yield predictions are well captured in several important major crop species through simulations (Lobell et al. 2011). These important crops are in need of adaptation investments to avoid catastrophic yield losses and to meet the food demand of a fast-increasing population. Drought is often accompanied by relatively high temperatures, which promote evapotranspiration and affects photosynthetic kinetics, thus intensifying the effects of drought and further reducing crop yields. It is anticipated that the occurrence of drought in many food-producing regions will increase significantly in response to climate change (Collins et al. 2008; Reynolds and Ortiz 2010).

Tolerance to drought is a complex quantitative trait controlled by several small effect genes or QTLs and is often confounded by differences in plants phenology (Barnabas et al. 2008; Fleury et al. 2010). To address the complexity of plant responses to drought, it is vital to understand the physiological and genetic basis of this response. Failure to understand the molecular mechanisms of seed yield stability has hampered both traditional breeding and the use of modern genetics in the improvement of drought tolerance of crop plants (Passioura 2010; Sinclair 2011).

Recent advances in crop physiology, systematic plant phenotyping and genomics have led to new insights in drought tolerance, thus providing crop breeders with greater knowledge of the gene networks and providing new tools for plant improvement to increase crop yield (Tuberosa and Salvi 2006). While plant physiology improves our understanding of the complex network of drought tolerance-related traits thus improving selection efficiency, molecular biology and genomics approaches identify the candidate genes and quantitative trait loci (QTLs) associated with these traits. While QTLs can be deployed in crop improvement through molecular breeding, candidate genes are the prime targets for generating transgenics using genetic engineering (Varshney et al. 2011). Identification of the “most appropriate” candidate genes along with selection of “most suitable promoters” and generation of a large number of events are critical for the development of desirable transgenics with enhanced drought tolerance using know-how knowledge (http://www.plantstress.com/; for a review see Luo 2010; Varshney et al. 2011). However, the expensive regulatory process and negative public perceptions of biosafety limit the application of genetic engineering approach, while there is a wider acceptance of products generated through molecular breeding (Vogel 2009; Farre et al. 2010; Varshney et al. 2011) and Targetted Induced Local Lesions in Genome (TILLING) (see Barkley and Wang 2008).

In the last decade, several important reviews of plant drought response and tolerance have been published (http://www.plantstress.com/files/Recent_Reviews/index.asp). The importance of multifaceted strategies including genetic engineering (Bhatnagar-Mathur et al. 2008; Yang et al. 2010), physiological approaches (Sinclair 2011) and genomics approaches (Tuberosa and Salvi 2006; Cattivelli et al. 2008; Ashraf 2010; Varshney et al. 2011) have been described in several crop species (e.g. maize, Tsonev et al. 2009; rice, Leung 2008; Bernier et al. 2008; wheat, Fleury et al. 2011; soybean, Manavalan et al. 2009; pearl millet, Yadav et al. 2011; canola, Wan et al. 2009). Also, the descriptions of molecular-physiological mechanisms of drought tolerance were outlined by several reviews (Bartels and Sunkar 2005; Maggio et al. 2006; Bressan et al. 2009; Charron and Quatrano 2009). In this review, we highlight the importance of drought tolerance, especially in a variable climate and discuss the recent progress made in the area of crop physiology for precise phenotyping and genomic approaches, such as identification and cloning of QTLs and identification of candidate genes associated with drought tolerance. In addition, new molecular breeding strategies such as marker-assisted recurrent selection (MARS) and genomic selection (GS) or genome-wide selection (GWS) are discussed as options to be integrated in crop improvement programmes for developing the next generation of drought-tolerant crops.

## The increasing importance of drought tolerance in variable climates

**Fig. 1:**
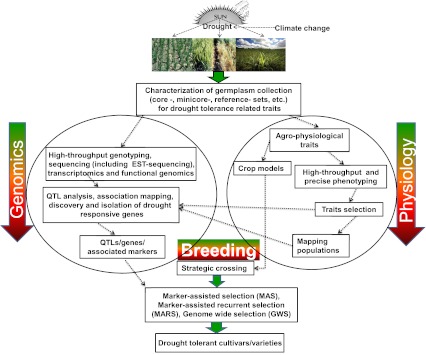
A holistic approach for integrating genomics, physiology and breeding approaches for developing the superior varieties with enhanced drought tolerance

The global water shortage caused by an increasing world population and worldwide climate change is considered as one of the major challenges facing agriculture today. The combination of continued impact of drought and high temperature impairs the photosynthesis during the day-time and increases the surface temperatures in the night, which in turn increase the photorespiratory losses and thus the productivity. The elevated greenhouse gas concentrations may lead to the general drying of the subtropics by the end of this century, thus creating widespread drought stress in agriculture [Inter-governmental Panel on Climate Change (IPCC) 2007]. This shortage of water may threaten sustainable crop farming, since agricultural activities account for 75 % of global water consumption and irrigation consumes over 90 % of water used in many developing countries (UNEP 2009; Yang et al. 2010). It is also anticipated that by 2030, developing countries will be most severely affected by climate change because: (a) climate change will have the greatest impact on the tropics and sub-tropics, (b) most of the predicted population growth to 2030 will occur in developing countries and (c) more than half of the workforce in developing countries is involved in agriculture (Reynolds and Ortiz 2010). In brief, the convergence of population growth and variable climate is expected to threaten food security on a worldwide scale. Relatively inexpensive and easier to adapt methods would be to switch crops or altering planting seasons according to predicted precipitation patterns and continued expansion of irrigation. However, worldwide occurrence of drought has become endemic due to climate change. This raises serious concerns and places huge responsibilities on the shoulders of scientists for developing “drought-suited varieties” through molecular breeding and genetically modified approaches. However, it is clear that the demand to produce sufficient major food crops (wheat, rice and maize) for the growing population has always been increasing. Hence, optimizing yield stability for these major crops and locally important crops is essential. Therefore, maintaining food security in this scenario will require systematic approaches (see later) including the use of drought-tolerant germplasm (Reynolds and Ortiz 2010). Recent advances in plant physiology, genomics and some future breeding strategies (Fig. 1) are believed to address the multigenic nature of abiotic stresses including drought tolerance.

## Addressing the complexity of plant response to drought

Among the various abiotic stresses that curtail crop productivity, drought is the most recalcitrant to breeding (Tuberosa and Salvi 2006), because plants use various mechanisms to cope with drought stress. In the past, drought tolerance breeding has been hindered by the quantitative inheritance of the trait and our poor understanding of the physiological basis of yield in water-limited conditions (Sinclair 2011), as well as by limitations in technology for systematic phenotyping.

The physiological dissection of complex traits like drought is a first step to understand the genetic control of tolerance and will ultimately enhance the efficiency of molecular breeding strategies. Developing and integrating a gene-to-phenotype concept in crop improvement requires particular attention to phenotyping and ecophysiological modelling, as well as the identification of stable candidate genomic regions through novel concepts of ‘genetical genomics’. Knowledge of both the plant physiological response and integrative modelling are needed to tackle the confounding effects associated with environment and gene interaction (Tardieu and Tuberosa 2010). To maximize the impact of using specific traits, breeding strategies requires a detailed knowledge of the environment where the crop is grown, genotype × environment interactions and fine tuning the genotypes suited for local environments. A physiological approach has an advantage over empirical breeding for yield per se because it increases the probability of crosses resulting in additive gene action for stress adaptation, provided that the germplasm is characterized more thoroughly than for yield alone (Reynolds and Trethowan 2007).

### Criteria for using physiological traits in breeding programmes

The use of physiological traits (PTs) in a breeding programme, either by direct selection or through a surrogate such as molecular markers, depends on their relative genetic correlation with yield, extent of genetic variation, heritability and genotype × environment interactions. For instance, in drought environments, osmotic adjustment, accumulation and remobilization of stem reserves, superior photosynthesis, heat- and desiccation-tolerant enzymes, etc. are important PTs. However, it is important to establish their heritability and genetic correlation with yield in target environments. Identification of drought-adaptive PTs and mechanisms is time consuming and costly; however, if successful, the benefits are likely to be substantial. The information on important PTs can be collected on potential parental lines involving screening of entire crossing block, or a set of commonly used parents, thus producing a catalogue of useful PTs. This information can be used strategically in designing crosses, thereby increasing the likelihood of transgressive segregation events, which bring together desirable traits. However, if enough resources are available, screening for PTs could be applied to segregating generations in yield trials, or any intermediate stage, depending on when genetic gains from selection are optimal (Reynolds 2002).

It is important to note that using specific traits, breeding strategies are effective only when these traits are properly defined in terms of the stage of crop development at which they are relevant, the specific attributes of the target environment for which they are adaptive and their potential contribution to yield (Reynolds and Trethowan 2007). The early escape from progressively intensifying moisture stress, through the manipulation of plant phenology, is the most commonly exploited genetic strategy used to ensure relatively stable yields under terminal drought conditions (Richards 1991). When significant genetic diversity for a physiological trait in a germplasm collection for the given species is established, it is imperative that the relevance of the trait as a selection criterion be determined.

### Conceptual framework for drought adaptation

The conceptual framework for yield drought adaptation by Passioura (1977) has three important drivers: (1) water uptake (WU), (2) water-use efficiency (WUE) and (3) harvest index (HI). These drivers stimulate trait-based breeding and genetic dissection of drought-adaptive mechanisms. Several traits have been found to be associated with the above yield component drivers. For WU, direct selection for variation in root characteristics is unfeasible; therefore, measurements associated with stomatal conductance like that of canopy temperature (CT) provide indirect indicators of water uptake by roots (see Reynolds and Tuberosa 2008). In addition, validation studies indicated that CT during peak stress periods was associated with ~50 % of the variation in water extraction in deep soil profiles and also with root length density (Reynolds et al. 2007a). For WUE, carbon isotope discrimination seems to be the best estimate and is based on higher affinity of the carbon-fixing enzyme (Rubisco) for the more common ^12^C isotope over the less common ^13^C. A lower discrimination value indicated higher WUE. Some other traits associated with WUE included spike photosynthesis in cereals, photoprotective mechanisms including antioxidant systems, regulation of water flow via aquaporins and signalling molecules such as abscisic acid (ABA) (see Reynolds 2002; Reynolds and Tuberosa 2008). Similarly, for HI, the extreme sensitivity of reproductive processes to drought may result in the reproductive failure, which is associated with low HI, and may eliminate benefits associated with favourable WU or WUE. Considering the overall contributions of these three yield drivers, WU is the most important for improving the yield potential (i.e. biomass) in drought environments, while stable HI is associated with higher yield potential (Blum 2009; Salekdeh et al. 2009). Storage of water-soluble carbohydrates (WSC) in the stem of small grain cereals and their subsequent remobilization to grain can directly influence HI, especially under post-anthesis stress. Translocation of soluble stem carbohydrates to the grain is one of the drought-adaptive traits that relates specifically to improved partitioning, though not to reproductive growth. Remobilization of stem reserves is associated with increased levels of ABA, which presumably is involved in the triggering of enzymes prerequisite to remobilization (Reynolds 2002). The yield potential (YP), expressed as a function of the light intercepted (LI) and radiation-use efficiency (RUE) (whose product is biomass), the partitioning of biomass to yield (the HI) and the focus of improving all the three components will be undertaken through complex physiological trait (PT)-based breeding.

A general model for drought adaptation of wheat was developed by the physiologists and breeders at CIMMYT that encompasses traits which possess a potential role in dry environments (Reynolds et al. 2005). In this model, some of the important traits included: (1) pre-anthesis growth, (2) access to water as a result of rooting depth or intensity that would be expressed by a relatively cool canopy (Reynolds et al. 2005), (3) water-use efficiency (WUE) as indicated by relatively higher biomass/mm of water extracted from the soil, transpiration efficiency of growth (TE = biomass/mm water transpired) indicated by C-isotope discrimination (Δ^13^C) of leaves, and WUE of spike photosynthesis associated with refixation of respiratory CO_2_, (4) photoprotection including energy dissipation, anti-oxidant systems and anatomical traits such as leaf wax. The model is used to assist in taking breeding decisions by permitting a strategic approach of accumulating drought-adaptive alleles by crossing parents with contrasting drought-adaptive mechanisms. Accumulation of soluble stem carbohydrates and their remobilization during the post-anthesis drought period help to supply surplus assimilates for grain growth during grain filling (Blum 1998). Similarly, root architecture that helps to have better access to soil moisture under drought enables heat-stressed crop canopies to meet high evaporative demand associated with hot, low-relative humidity environments, thus resulting in cooler canopies (Reynolds et al. 2000). Other traits impact either WUE or RUE depending on the environmental conditions (Reynolds and Trethowan 2007).

**Fig. 2:**
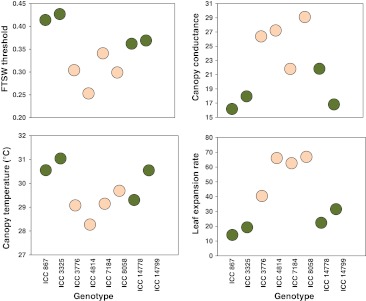
An example of involvement of several physiological traits for conferring terminal drought tolerance in chickpea. A set of eight chickpea genotypes including four tolerant (ICC 14799, ICC 867, ICC14788 and ICC 3325) and four susceptible (ICC 4814, ICC 8058, ICC 3776 and ICC 7184) to drought stress (*green filled circle* tolerant and *orange filled circle* sensitive) have been characterized for: canopy temperature (°C) and canopy conductance (mg H_2_O m^−2^ h^−1^) measured at 42 DAS under well-watered conditions; fraction of transpirable soil water (FTSW) threshold measured in plants exposed to progressive water stress and leaf expansion rate (LER; cm^2^ day^−1^), measured between 42 and 56 DAS. Susceptible genotypes tended to have lower canopy temperature and FTSW, but higher canopy conductance and LER as compared to tolerant genotypes (color figure online)

It is therefore crucial to target specific physiological mechanisms and to identify those traits most relevant to the patterns of drought stress found in the target environment. For example, in crops grown with residual soil moisture that experience terminal drought, such as chickpea (*Cicer arietinum*), genotypes with deeper, more profuse roots have an advantage through better water extraction deeper in the soil profile (Kashiwagi et al. 2005). In other crops also, deeper/profuse roots were found to increase plant access to water from deeper soil layers and support greater crop growth under drought conditions (Price et al. 2002a; Sinclair 2011). Therefore in several crops such as chickpea (Silim and Saxena 1993), wheat (Reynolds et al. 2007a) and rice (Yadav et al. 1997; Price et al. 2002a), deeper/profuse roots are targeted to improve grain yield under rainfed conditions. However, some recent studies (Zaman-Allah et al. 2011a, b) reported that selection for yield under terminal drought conditions was not essentially dependent on deeper/profuse root systems, but rather on several other critical traits that contribute to soil moisture conservation during late season water deficits. These traits include: (1) low leaf conductance under non-limited water conditions during the vegetative stage, which could be measured by a warmer canopy, (2) a low leaf expansion rate when soil moisture is still non-limiting for plant growth and a restriction of plant growth under progressive exposure to stress and (3) a higher fraction of transpirable soil water (FTSW) thresholds that reduce transpiration, thus avoiding rapid soil water depletion (Fig. 2). Several studies have shown that FTSW can be linked to variables describing plant water status such as midday leaf water potential, leaf relative water content and stomatal conductance (Sinclair and Ludlow 1986; Pellegrino et al. 2007), which are known to contribute to drought adaptation.

In addition to the above positive effects of the stay-green trait, enhanced remobilization of stored carbohydrates will lead to identify the important targets for enhancing seed sink strength under drought, thus helping to achieve yield stability under drought (for further details refer reviews by Sreenivasulu et al. 2007; Mittler and Blumwald 2010). Although, in general, photosynthesis is markedly reduced under drought stress, many dicot species are dependent on assimilates produced from current photosynthesis under drought and, therefore, exploring genotypes possessing efficient mechanisms of stay green will be beneficial. On the contrary, monocarpic cereal species seem to prefer assimilates produced prior to flowering (pre-anthesis assimilate), which is stored in the vegetative tissue, mainly in the stem in the form of various soluble sugars translocated through the trigger of remobilization events where ABA plays an important role (Yang and Zhang 2006; Zhang et al. 2009; Seiler et al. 2011). These traits were not all present in a single genotype, reflecting the complexity of drought tolerance and the need to pyramid several beneficial traits through plant breeding.

### Strategic trait-based crossing

The conceptual models of drought-adaptive traits have been found useful for accumulating complementary PTs in selected progeny. The key steps in this type of PT breeding include: (1) characterization of crossing block lines for stress-adaptive mechanisms, (2) strategic crossing among parents with different but potentially complementary PT expression, thus ensuring cumulative gene action in selected progeny, and (3) early generation selection (EGS) of bulks for canopy temperature (CT). This type of physiological characterization is used to assess variation and thereby increases the rates of genetic gains (Reynolds and Tuberosa 2008; Reynolds et al. 2009a). The main objective of strategic trait-based crossing is to accumulate traits that will be complementary for a given target environment. Under water-limited situations, traits that improve water uptake, water use efficiency and partitioning to yield, respectively, are likely to work synergistically to maximize productivity in the target environment (Passioura 1977; see Reynolds et al. 2009a). This has resulted in the distribution of advanced lines to rain-fed environments worldwide by the International Maize and Wheat Improvement Center (CIMMYT) and it has been confirmed that PT crossing results in cumulative gene action in selected progeny, resulting in increased yield under drought environments (Reynolds et al. 2005, 2007b; Reynolds and Tuberosa 2008). Characterization of candidate parents for better targeted crossing should have the highest priority in terms of physiological interventions in breeding for a number of reasons including: (1) since a large investment is needed in trait measurement and the information obtained can be used for many generations of crossing once the initial characterization has been made, (2) the number of lines in a crossing block are relatively small (~100/target environment) and the detailed characterization is even possible for traits which are relatively time-consuming, e.g. for traits like soil moisture depletion or stem carbohydrates.

The genetic gains in yield can be accelerated by incorporating complex PTs deterministically in modern plant breeding in addition to simply agronomic inherited traits like plant height, flowering time, resistance to prevalent diseases, quality parameters and yield based on multilocation trials (Braun et al. 2010; Reynolds et al. 2011). PT-based breeding approaches have been already implemented successfully in Australian breeding programmes (Rebetzke et al. 2009) as well as by CIMMYT, leading to international distribution of a new generation of elite drought-adapted lines (Reynolds et al. 2009b).

## Precise phenotyping for drought tolerance and related dynamic traits

After establishing the most suitable target trait for selecting grain yield under drought stress, the next step is to establish a high-throughput precision phenotyping platform for pinning down the source trait most tightly connected to yield (Tuberosa 2010). The precise phenotyping of drought-related PTs often requires the utilization of sophisticated and expensive techniques herein listed:

### Near-infrared (NIR) spectroscopy on agricultural harvesters

This method provides spectral information corresponding to the field plot in a single near-infrared spectrum, where physical and chemical characteristics of the harvested seed material are captured. By using calibration models (i.e. mathematical and computational operations that relate the spectral information with phenotypic values), several traits can be determined on the basis of a single spectrum (dry matter, protein, nitrogen, starch and oil content, grain texture and grain weight, etc.; Montes et al. 2007; Wiley et al. 2009; Hacisalihoglu et al. 2010). The use of NIR spectroscopy on agricultural harvesters provides indexing of grain characteristics. In contrast to conventional sample-based methods, NIR spectroscopy on agricultural harvesters secures a good distribution of measurements within plots and covers substantially larger amounts of plot material (Welle et al. 2003), thus reducing sampling error and providing more representative measurements of the plot material in terms of homogeneity.

### Canopy spectral reflectance (SR) and infrared thermography (IRT)

Spectral reflectance of plant canopy is a non-invasive phenotyping technique that enables several dynamic complex traits, such as biomass accumulation, to be monitored with high temporal resolution (Montes et al. 2007). It has many advantages including easy and quick measurements, integration at the canopy level and additional parameters can also be measured simultaneously via a series of diverse spectral indices like photosynthetic capacity, leaf area index, intercepted radiation and chlorophyll content. Therefore, canopy reflectance is considered as one of the valuable tools for high-throughput phenotyping (Montes et al. 2007; Chapman 2008; Gutierrez et al. 2010). In soybean, canopy reflectance indices have been already used with great promise for measuring the effects of increasing atmospheric CO_2_ and O_3_ on soybean canopies (Gray et al. 2010).

Investigations at the individual plant level under well-controlled environmental conditions showed that spectral reflectance could be used to: (1) estimate the effects of environmental perturbations, such as changing atmospheric composition, on canopy structure and function (Gray et al. 2010), (2) monitor plant photosynthetic pigment composition and (3) assess plant water status and detect abiotic or biotic plant stresses (Chaerle and van der Straeten 2000; Gutierrez et al. 2010).

Plant water status as determined by plant water content or water potential (Jones 2007; Jones et al. 2009) integrates the effects of several drought-adaptive traits. Several methods are used to determine crop water content, including leaf water potential, leaf stomatal conductance and canopy temperature (CT), which is the relative measure of water flow associated with water absorption from the soil under water deficit (Reynolds et al. 2007a, b).

In addition to the above, one of the most commonly used indirect techniques for measurement of these variables is thermal infrared imaging, or infrared thermography, which involves the measurement of leaf or canopy temperature. Plant canopy temperature is a widely measured variable that is closely related to canopy conductance at the vegetative stage (Zaman-Allah et al. 2011a) and therefore provides insight into plant water status. In any given environment, stomatal variation is the dominant cause of changes in canopy temperature (Jones 2004). Although thermal imaging does not directly measure stomatal conductance, it has become a high-throughput tool for estimating differences in stomatal conductance (Merlot et al. 2002). Thermal infrared imaging for estimating conductance can be used at the whole plant or canopy level over time.

### Magnetic resonance imaging (MRI) and positron emission tomography (PET)

These two methods are used at the Jülich Plant Phenotyping Centre, Germany (Heike Schneider, personal communication) to investigate root/shoot systems growing in sand or soil which allow to assess structure, transport routes and the translocation dynamics of recently fixed photoassimilates labelled with short-lived radioactive carbon isotope (δ^11^C). Quantitative MRI and PET data not only help to study differences between species, but also provide a phenotype within a species, the growth pattern, water relations and/or translocation properties of assimilates (Jahnke et al. 2009). Therefore, the MRI–PET combination can provide new insights into structure–function relationships of intact plants. It also allows monitoring of dynamic changes in plant properties, which have previously not been possible to assess systematically, thus improving our understanding of plant performance (such as resource use efficiency or biomass production).

### Nuclear magnetic resonance (NMR)

The short half-life of δ^11^C (only 20 min) limits the utility of MRI to study source–sink relationships in minute structures such as developing seeds (Jahnke et al. 2009). NMR provides an alternative in vivo detection platform using ^1^H NMR and utilizes the signal emitted by protons associated with carbon nuclei, thereby sucrose and water movement may be imaged and quantified (Sardans et al. 2010; Melkus et al. 2011). Therefore, NMR technology is employed with ^13^C/^1^H double-resonant high-resolution coil to achieve better resolution for monitoring the structure of tissues like seeds through *non*-*invasive* visualization, mapping water movements and monitoring of sucrose allocation using ^13^C-labelled sucrose (Neuberger et al. 2008; Melkus et al. 2011).

### Integrative platforms

One of the high-throughput integrated phenotyping platforms that includes the pipeline of imaging, image processing automatization and data handling modules was developed by LemnaTec, a German company (http://www.lemnatec.com). The platform has the capacity to measure almost unlimited sets of parameters easily, allows comprehensive screening and provides statistics on various plant traits in a dynamic way. Depending on the degree of automatization, plants are manually placed in the Scanalyzer 3D or transported on conveyor belts directly from the greenhouses to the imaging chambers. Such chambers provide top and side imaging of both shoot and root systems to quantify plant height/width, biomass and plant architecture. Application of different camera and acquisition modes—from visual light to near infrared (NIR/SWIR), infrared (IR) and fluorescence imaging—opens new perspectives for visualization using non-destructive quantification. The key application is in the fast developing domain of plant functional genomics. These automated systems will increase our understanding of plant growth kinetics and help improve plant models for systems biology or breeding programmes.

In summary, the techniques and platforms mentioned above will greatly improve the phenotyping accuracy and throughput, thus contributing to a better elucidation of the genetic control of complex drought tolerance traits in plants. However, many of the techniques discussed above are applied to plants grown under controlled conditions that may not reflect field environment or can only be used to assess a limited number of genotypes due to high costs and/or practicality. Therefore, to overcome this problem, multi-tiered selection screens, where a simple but less accurate screen allows large number of genotypes to be evaluated (first screen), followed by tiers of more sophisticated screens of decreasing numbers of genotypes have been proposed (Sinclair 2011, Fig. 3). A three-tiered sequence of physiological screens have been already used to identify candidate parental genotypes for use as parents in breeding programs for some key traits like nitrogen fixation activity during soil water deficit in soybean (Sinclair et al. 2000). Furthermore, bringing integrative phenotyping technology, such as that developed by LemnaTec, from the controlled environments to the field will improve the assessment of plant responses to drought while enabling high-throughput screening and generating comprehensive and accurate phenotypic data.

**Fig. 3:**
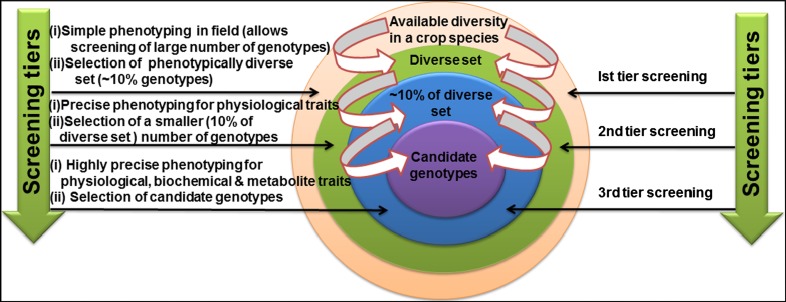
An overview of three-tier screening of germplasm collection for traits related to drought tolerance. Three screening tiers are shown on the *right side* and the procedure of selection of germplasm followed in each tier of screening is provided on the *left side*

## Molecular dissection of drought tolerance

In several genetic studies, drought tolerance has been found to be a complex quantitative trait controlled by a large number of minor genes/QTLs (Fleury et al. 2010; Ravi et al. 2011). Recent advances in genome mapping and functional genomics technologies have provided powerful new tools for molecular dissection of drought tolerance (Worch et al. 2011). The molecular markers and/or candidate genes identified provide a better understanding of the molecular basis of drought tolerance and, once validated, can be used in molecular breeding.

### QTL discovery for drought tolerance-related traits

Traditional QTL mapping involves: (1) development of mapping populations segregating for drought tolerance-related traits, (2) identification of polymorphic markers, (3) genotyping of the mapping populations with polymorphic markers, (4) construction of genetic maps, (5) precise phenotyping for drought tolerance-related traits, as mentioned above, and (6) QTL mapping using both genotypic and phenotypic data. This process is commonly called linkage mapping/linkage analysis-based QTL mapping (see Chamarthi et al. 2011). During the past decade, a large number of studies involving linkage mapping have been conducted in several crops to identify QTLs linked to drought tolerance (for reviews see, Cattivelli et al. 2008; Fleury et al. 2010). However, linkage mapping-based QTL mapping does not provide precise information on QTLs because of inherent limitations associated with each mapping population. Some of these limitations are summarized by Myles et al. (2009) and include: (1) insufficient time for recombination to occur and shuffle the genome into small fragments, and as a result the QTLs identified are generally localized to large genomic regions/chromosomal segments, (2) insufficient phenotypic variation for the trait present in the mapping population and (3) segregation of different QTLs for the same trait in different mapping populations.

To overcome some of above constraints, linkage disequilibrium (LD)-based association mapping, initially used in human genetics, has been suggested as an alternative approach for QTL mapping in crop species (e.g. Myles et al. 2009; Rafalski 2010). The association mapping (AM) approach involves: (1) selection of a diverse association panel/group of individuals from a natural population/germplasm collection, (2) precise recording of phenotypic data on the panel, (3) candidate gene sequencing or high-density marker genotyping of the panel, (4) study of population structure (the level of genetic differentiation among groups within the selected population) and kinship (coefficient of relatedness between pairs of each individual within the population) and (5) association analysis based on information gained through population structure, kinship, and correlation of phenotypic and genotypic/haplotypic data. AM offers several advantages over bi-parental linkage mapping and these include: (1) exploitation of all the recombination events that took place during the evolutionary history of a crop species, resulting in much higher mapping resolution, (2) less time required in mapping QTL as there is no need to develop a specialized mapping population, rather a natural germplasm collection of a crop species is sufficient, (3) cost-effectiveness because the same AM panel and genotyping data can be used for mapping of different traits, (4) populations can be structured to avoid randomly generated lines (recombinant inbred lines; RILs), many of which express substandard agronomic type and (5) a higher number of alleles can be sampled compared to linkage mapping where only two alleles are usually surveyed (in apple or potato where heterozygous parental lines are used, more than two alleles/locus can be present). Markers linked to drought tolerance traits, identified using AM, have been reported in wheat (Sanguineti et al. 2007; Maccaferri et al. 2011), barley (Ivandic et al. 2003; Baum et al. 2007; Varshney et al. 2012) and maize (Lua et al. 2010). However, obtaining a clean set of reproducible phenotypic data of drought tolerance from a larger germplasm collection for AM studies remains an open challenge even in the era of phenomics-driven technology.

In summary, QTLs for drought tolerance have been identified for several major and important crop species like rice, maize, wheat, barley, sorghum, pearl millet, soybean and chickpea (see Table 1). These QTLs were identified for a variety of important traits including: (1) yield and yield-contributing traits under water-deficit conditions (in the case of wheat, maize, rice, soybean and pearl millet), (2) physiological responses including water-soluble carbohydrates, carbon isotope ratio, osmotic potential, chlorophyll content, flag leaf rolling index, grain carbon isotope discrimination, relative water content, leaf osmotic potential, osmotic adjustment, chlorophyll and chlorophyll fluorescence parameters to drought stress (in the case of wheat, maize and rice), (3) flowering time including anthesis to silking interval (in maize), (4) root traits (rice, maize, wheat, soybean and chickpea), (5) stay green (sorghum) and (6) nitrogen fixation (soybean). However, so far QTL studies on the impact of drought on grain quality have not been documented. While some key QTL studies in some crop species have been summarized in Table 1, an updated compilation of mapped QTL and major genes associated with abiotic stress tolerance including drought tolerance in crop plants is available at PLANTSTRESS site (http://www.plantstress.com/biotech/index.asp?Flag=1). Most of the identified QTLs for drought traits explain a relatively small portion of total phenotypic variation. As a result, their direct deployment in breeding programmes through marker-assisted selection (MAS) may not be very effective.

**Table 1:** Summary of QTLs identified for drought tolerance-related traits in some major crop species

Crop	Traits studied	Number of QTLs	Chromosome/linkage group	Phenotypic variation explained (PVE %)	Reference
Rice	Grain yield	1 (*qtl12 .1*)	12	51.0	Bernier et al. (2007, 2009)
	Grain yield	2	2, 3	13.0–31.0	Venuprasad et al. (2009)
	Relative growth rate and specific water use	7	2, 4, 5, 6, 7, 8	10.0–22.0	Kato et al. (2008)
	Coleoptile length and drought resistance index	15	All except 3, 8, 11	4.9–22.7	Song-ping et al. (2007)
	Basal root thickness and 100-grain weight	2	4, 6	20.6–33.4	Li-Feng et al. (2007)
	Grain yield and other agronomic traits	77	All except 12	7.5–55.7	Lanceras et al. (2004)
	Root traits	18	All chromosomes	1.2–18.5	Ping et al. (2003)
	Root and related traits	42	All chromosomes	6.0–24.4	Courtois et al. (2003)
	Water stress indicators, phenology and production traits	47	All except 5	5.0–59.0	Babu et al. (2003)
	Drought avoidance	17	All except 9	4.4–25.6	Price et al. (2002b)
	Osmotic adjustment	1 (*OA*_*70*_)	8	Major	Lilley et al. (1996)
Maize	Yield components and secondary traits	81	–	0.1–17.9	Messmer et al. (2009)
	Grain yield and yield components	20	1, 2, 3, 5, 7, 8, 9	4.1–31.3	Xiao et al. (2005)
	Root characteristics, drought tolerance index and yield	56	All chromosomes	6.7–47.2	Tuberosa et al. (2002)
	Leaf ABA	1 (*L*-*ABA*)	2 (bin 1.03)	32.0	Tuberosa et al. (1998); Landi et al. (2005)
	Grain yield and yield components	46	All except 10	4.0–12.9	Ribaut et al. (1997)
	Anthesis–silking interval	6	1, 2, 5, 6, 8, 10	48 (total)	Ribaut et al. (1996)
Wheat	Agronomic, phonological and physiological traits	104	1A, 1B, 1D, 2B, 3A, 3B, 4A, 4B, 4D, 5A, 5B, 6A, 6B, 7A, 7B, UA-b	11.2–33.5	Pinto et al. (2010)
	Morpho-physiological traits	110	All 14 chromosomes	0.8–42.4	Peleg et al. (2009)
	Grain yield and growth traits	42	1A, 1B, 2A, 2B, 3A,3B, 4A, 4B, 5A, 6B, 7A and 7B	3.4–53.9	Maccaferri et al. (2008)
	Water-soluble carbohydrates and associated traits	48	All chromosomes except 2B, 3D, 4D, 5D, and 6D	1.1–7.6	Yang et al. (2007)
	Grain yield and yield components under drought	1	4AL	12.0–41.0	Kirigwi et al. (2007)
	Yield and growth traits	16	1B, 1D, 2B, 3A, 4A, 4B, 4D, 5A, 5B, 6A, 6B, 6D, 7A, 7B	–	Mathews et al. (2008)
	Stem reserves mobilization	3	2D, 5D, 7D	21.1–42.3	Salem et al. (2007)
Barley	Drought-related morphological and physiological traits	18	1H, 2H, 3H, 4H, 5H, 6H, 7H	14.3–57.5	Chen et al. (2010)
	Chlorophyll and chlorophyll fluorescence parameters	5	1H, 2H, 4H, 6H, 7H	6.2–13.6	Guo et al. (2008)
	Yield and growth traits	42	All chromosomes	6.5–36.9	von Korff et al. (2008)
	Drought-related morphological and physiological traits	68	IH, 2H, 3H, 4H, 5H, 6H, 7H	4.0–16.0	Diab et al. (2004)
	Yield and other agronomic traits	74	All chromosomes	1.4–84.8	Baum et al. (2003)
	Relative water content	6	2H, 5H, 6H, 7H	6.8–11.5	Teulat et al. (2003)
	Grain carbon isotope discrimination	10	2H, 3H, 6H, 7H	–	Teulat et al. (2002)
	Osmotic adjustment (OA) and related traits	22	1H, 2H, 4H, 5H, 7H	5.0–20.0	Teulat et al. (2001a)
	Grain yield and agronomic traits	56	All chromosomes	5.7–23.6	Teulat et al.(2001b)
	Osmotic adjustment (OA) and related traits	12	1H, 2H, 5H, 6H	5.8–26.7	Teulat et al. (1998)
Sorghum	Stay green	1 (*Stg2*)	A	53.5	Sanchez et al. (2002)
	Stay green	10	A, C, D, E, G, H	5.1–26.3	Haussmann et al. (2002)
	Stay green	9	A, B, C, D, E, F, G, J	9.9–22.6	Kebede et al. (2001)
	Stay green	8	A, D, E, J	9.1–32.6	Subudhi et al. (2000)
	Stay green	4	A, D, J	13.0–30.0	Xu et al. (2000)
	Stay green	5	B, G, I	10.7–14.1	Tao et al. (2000)
	Stay green and maturity	9	A, B, D2, G, I1, I2, J	7.7–47.5	Crosta et al. (1999)
Pearl millet	Grain yield and related traits	20	1, 2, 3, 4, 5, 6, 7	11.6–57.2	Bidinger et al. (2007)
	Grain yield and other physiological traits	46	1, 2, 3, 4, 6, 7	8.4–57.2	Yadav et al. (2002)
Soybean	Yield and wilting	6	D2, F, F2	–	Monteros et al. (2006)
	Leaf wilting	1	K	17.0	Bhatnagar et al. (2005)
	Yield	1	C2	7.0	Specht et al. (2001)
	Water use efficiency	7	L	8.0–14.0	Mian et al. (1998)
	Water use efficiency	5	G, H, J, C1	5.0–13.2	Mian et al. 1996)
Common bean	Yield and yield component traits	49	All except LG1	7.0–31.0	Blair et al. (2012)
	Yield and yield component traits	9	–	–	Schneider et al. (1997a)
Tomato	Seed germination	4	1, 8, 9, 12	Major effects	Foolad et al. (2003)
	Water use efficiency	3	Undetermined	–	Martin et al. (1989)
Cotton	Productivity and physiological traits	79	1, 2, 4, 5, 6, 9, 10, 11, 12, 13, 14, 15, 18, 20, 22, 25	1.7–23.7	Saranga et al. (2004)
	Productivity and physiological traits	16	2, 3, 4, 5, 6, 7, 9, 14, 15, 18, 22, 25	4.1–16.2	Saranga et al. (2001)

### QTL cloning for drought tolerance-related traits

In general, QTLs identified through linkage mapping-based approaches have low resolution and have been located in 10–20 cM intervals. The support interval of the QTL may also span several hundreds of genes and identifying the right candidate gene(s) with causal effect on the trait is like finding a ‘needle’ in the ‘genomic haystack’. Therefore, to identify the causal gene(s), positional cloning of QTLs have been undertaken in several crop species (Salvi and Tuberosa 2005; Tuberosa and Salvi 2006). QTL cloning, in general, involves the following steps: (1) delimiting the QTL region by using a large mapping population (>1,500 plants) derived from a cross between two NILs for the target QTL, (2) identifying the contig covering the QTL region by screening the closely linked molecular markers with a large insert library like BAC (bacterial artificial chromosome) library, (3) sequencing the contig and candidate gene identification based on sequence data and (4) validating the effect of candidate gene(s) on phenotype.

Although many reports are available on cloning of QTLs associated with different traits (see Salvi and Tuberosa 2007), there are few reports addressing QTL cloning for drought tolerance traits. For instance, a major flowering time QTL “*Vgt1*” associated with drought tolerance has been cloned in maize (Salvi et al. 2002, 2007). Recently, the gene encoding ATP-binding cassette (ABC) subfamily G (*HvABCG31*) full transporter was cloned from *eibi1* mutation responsible for leaf water conservation in wild barley and rice (Chen et al. 2011). Newer genomics approaches like association mapping and next-generation sequencing (NGS) hold great promise for accelerating QTL cloning of drought tolerance-related traits. The cloning of drought tolerance QTLs provides an opportunity to validate candidate genes that can be used to develop transgenic plants, not only in the original crop species but also in other crop species.

### Identification of genes associated with drought tolerance

The significant advances made in the model plant systems of major crop species provide an opportunity to identify candidate genes associated with drought tolerance. Some approaches are discussed in the following section:

#### Candidate genes (CGs) from model plant species

Genome sequences have recently become available for several model and major plant species (Feuillet et al. 2010). Genome annotation, molecular physiological as well as functional genomics studies undertaken in model and/or major crop species provide evidence of the candidate genes (CGs) involved in conferring drought tolerance. The CGs can be: (a) genes involved in cell protection under drought stress (e.g. proteins involved in osmotic adjustment, degradation, repairs, detoxification and structural adaptations) and/or (b) genes involved in regulation of other genes involved in the drought response (protein kinases and transcription factors such as DREB, bZIP, MYB, etc.). Knowledge of the CGs responsible for drought tolerance is useful for understanding the functional basis of drought tolerance and assists in their subsequent use, once they are validated, in molecular breeding through MAS. For instance, a set of nearly 30 important candidate genes associated with drought tolerance have been compiled by Sehgal and Yadav (2010). Validation of the CGs, an important and essential step before they can be deployed, can be undertaken using several approaches including integration with QTL maps, association mapping, expression analysis using qRT-PCR, allele mining and TILLING. Several of these approaches in relation to breeding programme applications are discussed by Varshney et al. (2005). One such example is the mapping of two CGs (*OsEXP2* and *EGase*) involved in cell expansion within the expected intervals of QTL for root traits in rice (Zheng et al. 2003). Similarly, 16 CGs associated with drought tolerance were included in the integrated QTL and physical map of rice (Wang et al. 2005). However, candidate genes have not delivered as much as anticipated for crop breeding, especially for drought tolerance.

#### Transcriptomics and functional genomics

Transcriptomics and functional genomics have been used extensively in recent years to better understand the stress-responsive mechanisms in crop plants. The candidate genes associated with drought tolerance mechanisms have been identified, characterized and assessed for their transcriptome responses using whole-genome sequencing or through micro-array technologies. The generation of ESTs from either normalized or non-normalized cDNA libraries from drought-challenged tissues of drought-responsive genotypes is one of the most common approaches for isolation of drought-responsive candidate genes. A large number of drought-responsive genes have been generated in several crop species. In rice, normalized cDNA libraries from drought-stressed seedlings led to the identification of novel genes that were abundantly expressed under drought stress and so far dozens of rice genes have been identified as drought responsive (Reddy et al. 2002; Hadiarto and Tran 2011 and references therein). Similarly, a survey of all the publicly available ESTs in various cereal crops including barley, maize, rice and wheat has led to the identification of drought stress-responsive genes in these species (Sreenivasulu et al. 2004, 2007; Kathiresana et al. 2006). In case of chickpea, twofold transcriptional changes were observed for 109 genes under drought (Mantri et al. 2007) and >220 (70 %) drought-tolerant unique ESTs were identified by Jain and Chattopadhyay (2010). In addition, 11,904 drought-responsive ESTs were generated earlier at ICRISAT for chickpea (Varshney et al. 2009a). This study was further extended by the National Research Centre on Plant Biotechnology (NRCPB) in India and 5,494 high-quality drought-responsive ESTs were isolated using suppression subtraction hybridization (SSH) of drought-challenged root and shoot tissues (Deokar et al. 2011). Such studies provide an important resource for marker development and also act as resource for the identification and selection of candidate genes (both up- and down-regulated) associated with drought tolerance. Although bioinformatics analysis (e.g. BLASTX) of such ESTs can help to identify the most promising EST/gene(s), it is essential to prove the function of the most promising genes using wet laboratory experiments such as qRT-PCR.

Another approach to identify candidate genes is transcript profiling that involves analysis of differential gene expression in the given tissue at different time points after exposure of the plant to drought stress or between drought-tolerant and susceptible genotypes (Hazen et al. 2003; Shinozaki et al. 2003; Micheletto et al. 2007; Hampton et al. 2010). However, it is important to target the right tissue and the precise stage of tissue in addition to the dynamics (i.e. timing and intensity) of the stress treatment imposed to mimic drought conditions for isolation of RNA for use in transcriptomics studies (Talamè et al. 2007). Instead of using genotypes with different genetic background, near-isogenic lines (NILs), which differ only in the target trait, are the ideal genetic material that ensures differentially expressed genes are linked to the trait and not to the genetic background. More recently, it was demonstrated that miRNAs are also involved in drought stress response/tolerance in crop plants including rice (Zhou et al. 2010) and soybean (Kulcheski et al. 2011) and their validation revealed their possible involvement in drought tolerance (Kulcheski et al. 2011).

Several platforms have become available for transcript profiling: (a) PCR-based differential display PCR (DDRT-PCR) analysis (Liang and Pardee 1992), (b) cDNA–Amplified Fragment Length Polymorphism (cDNA–AFLP) analysis (Bachem et al. 1996), (c) cDNA and oligo-nucleotide microarrays (Sreenivasulu et al. 2010) and (d) digital expression analysis based on counts of ESTs (Varshney et al. 2009a; Raju et al. 2010). SuperSAGE (Matsumura et al. 2003, 2010), an improved version of the serial analysis of gene expression (SAGE) technique, has been also successfully applied in several crop plants including chickpea for expression analysis of ~80,000 transcripts from unstressed and drought-stressed roots (Molina et al. 2008). However, with the advent of NGS technology (Varshney et al. 2009b), the sequence-based transcriptome analysis is in many ways considered superior to microarrays in orphan crops where genome sequence information is lacking, since the sequencing-based method is real time, digital and highly accurate. Therefore, it is anticipated that microarrays may soon be replaced by sequencing-based digital gene expression analysis (Shendure 2008; Varshney et al. 2009b). The application of NGS technologies to gene expression analysis has catalysed the development of techniques like Digital Gene Expression TAG (DGE-TAG), DeepSAGE (Nielsen et al. 2006, 2008) and RNA-Seq (Marioni et al. 2008; Nagalakshmi et al. 2008). RNA-seq based on NGS technologies has several advantages for examining transcriptome fine structure including detection of allele-specific expression and splice junctions (Malone and Oliver 2011) and may allow direct high-throughput sequencing of RNA from the stress (e.g. drought)-challenged tissues of different genotypes. Such transcript profiling (including RNA-seq) based on drought-tolerant and drought-sensitive genotypes can identify candidate genes associated with drought tolerance that can be used as genic molecular markers (GMMs) and integrated into genetic/QTL maps (Hiremath et al. 2011). It is possible that some candidate genes identified as above may be associated with QTLs for drought tolerance traits. In such cases, a genetical genomics approach that involves quantitative analysis of transcript profiling of the candidate genes can provide the e(xpression) QTLs for drought tolerance-related traits (Varshney et al. 2005). In case eQTLs are found in the *cis*-condition, then the candidate gene-based molecular markers should act as the functional and diagnostic markers for the respective traits (Potokina et al. 2008). It is anticipated that NGS-based transcript profiling should be routinely used for major crop species in the identification of candidate genes for drought tolerance and for subsequent use in genetical genomics or molecular breeding.

## Modern breeding approaches for developing superior germplasm for drought tolerance

Once the candidate genes or markers associated with QTLs for drought tolerance are identified, the next step is their deployment in breeding practices. Some of these approaches are discussed below.

### Marker-assisted backcrossing (MABC)

When the QTLs identified for drought tolerance traits contribute higher phenotypic variation, they are considered major QTLs. These QTLs, after validation in desired germplasm, can be used for introgressing drought tolerance from the donor genotypes (generally used for identification of the QTL for the trait) into elite, less drought-tolerant cultivars or breeding lines (recipient parents) without transfer of undesirable or deleterious genes from the donors (linkage drag). The process is commonly referred to as marker-assisted backcrossing (MABC). Superior lines or cultivars are developed that contain only the major gene/QTL from the donor parent, while retaining the whole genome of the recurrent parent (Hospital 2003; Varshney and Dubey 2009; Gupta et al. 2010). Although MABC has been used extensively for introgressing resistance to biotic stresses, only a few reports are available on the use of MABC to develop the superior lines/varieties for drought tolerance (Table 2). For instance, MABC has been used to introgress root trait QTLs in the elite rice cultivars IR64 and Kalinga III (Shen et al. 2001; Steele et al. 2006). By using these MABC products, a variety namely “Birsa Vikas Dhan 111 (PY 84)” was developed and released in Jharkhand State of India (Steele et al. 2007). In this example, MABC was used to transfer multiple QTLs for improved root growth under drought conditions. Similar work was done in maize to introgress favourable alleles at five target regions that influence the expression of yield components, flowering traits (including anthesis–silking interval (ASI)) and increased grain yield under water-limited conditions (see Ribaut and Ragot 2007). Backcross-derived lines differing in the parental alleles (Os420 and IABO78) at a major QTL (*root*-*ABA1*) have also been developed in maize (Tuberosa et al. 1998; Sanguineti et al. 1999) and a very strong and consistent effect of this QTL on leaf ABA concentration (L-ABA) across different water regimes has been confirmed in subsequent studies (Giuliani et al. 2005; Landi et al. 2005, 2007). Field evaluation conducted under well-watered and water-stressed conditions in two consecutive seasons indicated that each pair of *root*-*ABA1* backcross-derived near isogenic lines differed significantly and markedly for L-ABA, thus confirming the effectiveness of MAS (Landi et al. 2005). Similarly, a major QTL for improved grain yield in pearl millet under terminal drought stress when transferred into a drought-sensitive genotype showed a consistent grain yield advantage (Serraj et al. 2005). Key reports on MABC for drought tolerance have been compiled in Table 2.

**Table 2:** Some examples of marker-assisted selection (MAS) for drought tolerance in crop plants

Crop	Trait improved	No. of genes/QTL transferred	Reference
Rice	Yield and grain quality under drought	Multiple QTL	Steele et al. (2006, 2007)
Cotton	Drought tolerance-related traits	7 QTLs	Levi et al. (2009)
Common bean	Drought tolerance-related traits	Multiple QTL (9 RAPD markers)	Schneider et al. (1997b)

The relatively low success of MABC for improving drought tolerance can be attributed to the complex nature of drought. In many instances, the expression of drought tolerance is controlled by minor main-effect QTLs or epistatic QTLs. For instance, QTLs with ~10 % phenotypic variation for drought tolerance have been identified in maize (Xu et al. 2009), groundnut (Ravi et al. 2011), etc. These studies highlight the need to transfer several QTLs/genes to achieve a significant impact, assuming additive variance, and this may require unmanageable population sizes (Ribaut et al. 2010).

### Marker-assisted recurrent selection (MARS)

To overcome the limitations of MABC, particularly when multiple QTLs control the expression of a complex trait, the MARS approach, which involves intermating selected individuals in each selection cycle, has been recommended (Eathington et al. 2007; Ribaut and Ragot 2007; Bernardo 2008). It generally involves the use of an F_2_ base population, and can be used in self-pollinated crops like wheat, barley and chickpea for developing pure lines with superior per se performance (for more details, see Bernardo 2008). MARS has the additional advantage of overcoming the limitation of inadequate improvement in the frequency of superior alleles in F_2_ enrichment, since MAS is practised in each cycle following intermating to improve the frequency of favourable alleles (Eathington et al. 2007). The successful use of MARS has been reported in sweet corn (Edwards and Jonson 1994), sunflower and soybean (Eathington et al. 2007). In case of wheat, MARS for water use efficiency is being exercised under an Indo-Australian project involving partners from DWR, Karnal, PAU Ludhiana, IARI, New Delhi and Australia. Generation Challenge Programme (GCP) also launched a challenge initiative to improve heat/drought tolerance in wheat through MARS approach involving the Indian Agricultural Research Institute (IARI), New Delhi, India, Chinese Academy of Agricultural Sciences (CAAS), China, and partners from Australia (http://www.generationcp.org/ci_feb_2010_launch_meeting_feature). Similar MARS breeding programmes are being conducted at several other international institutes including ICRISAT, the French Centre for International Agricultural Research (CIRAD) and University of California-Riverside, USA for improving drought tolerance in chickpea, sorghum and cowpea, respectively (see Kulwal et al. 2011).

### Genome-wide selection (GWS)

Genome-wide selection (GWS) or genomic selection (GS) is another important approach to develop superior germplasm lines with overall excellent performance in a target environment.

Genome-wide marker genotyping is used for GWS rather than selected markers showing significant associations (as in case of MARS) with the traits of interest. In summary, individuals in a phenotyped population (generally referred to as the ‘training population’) are genotyped using genome-wide markers and breeding values of alternative alleles of all the markers are fitted as random effects in a linear model. Individuals in subsequent recurrent selection generations are then selected based purely on the sum of those breeding values [genomic estimated breeding value (GEBV); Meuwissen et al. 2001]. Therefore, GWS reduces the frequency of phenotyping and similarly also increases annual gains from selection by reducing cycle time (Rutkoski et al. 2010). Several groups have recently started exploring the GWS approach in both self- and cross-pollinated crops with some modifications for both types of crops (Bernardo 2010). The success of the GWS approach is dependent on the availability of a diverse and representative training population. Furthermore, the phenotyping of the training population is crucial and additional lines should be integrated over time to increase the effectiveness and relevance of the gene effect estimates. This approach has been recently used to improve durable stem rust resistance in wheat (Rutkoski et al. 2010) and eventually could be systematically explored to bring different components of multigenic drought tolerance using the GWS approach.

## Lessons learnt and future outlook

It is evident that precise phenotyping is essential to screen larger core collection/mapping population for identifying the most appropriate QTL and candidate genes for use in plant breeding. A number of phenotyping approaches are available and this area of research is currently referred to as ‘phenomics’. Nevertheless, re-integration of the pieces of the ‘phenomics’ puzzle into a comprehensive and relevant crop improvement framework of ‘seed yield stability’ will involve crop modelling (Tardieu and Tuberosa 2010). The combination of phenomics and modelling offers great potential to rapidly assess the value of certain traits on plant performance. The use of models to understand gene-to-phenotype relationships provides an efficient platform for a new and creative interaction between genetics–genomics and crop physiology (Edmeades et al. 2004). To meet the real-world challenge of increased crop production, the information available from functional genomics and systems biology needs to be integrated at the crop level; thereby, crop physiology will have a fundamental role in achieving this goal. A new generation of crop models combined with systems biology studies should enable us to significantly narrow the gap between genes and complex phenotypes by predicting the field performance of crop genotypes (Yin et al. 2004). Crop models will significantly contribute to higher level of integration by directly linking physiological processes to complex crop phenotypes within the scope of source–sink relationships. Similarly, recent advances in genomics make it possible to not only conduct large-scale and high-throughput marker genotyping, but also sequence or re-sequence the genomes of germplasm collections, thus facilitating the identification of QTLs and candidate genes associated with drought tolerance. While commonly used MABC has not been very effective in developing superior lines for drought tolerance, modern breeding approaches such as MARS and GWS are powerful tools for pyramiding multiple QTLs for drought tolerance or introgressing multiple complex traits such as heat tolerance in addition to drought tolerance.

In summary, it is essential to integrate crop physiology, genomics and breeding approaches to dissect complex drought tolerance traits, understand the molecular basis of drought tolerance and develop the next-generation crops for our changing climate. Although work is ongoing in some major crops, it is anticipated that integrated physiology, genomics and breeding approaches will be initiated/accelerated in the so-called orphan crops that are important for food security in many developing countries.
